# Contributions of *PARP‐1* rs1136410 C>T polymorphism to the development of cancer

**DOI:** 10.1111/jcmm.16027

**Published:** 2020-10-27

**Authors:** Hunian Li, Yongjiu Zha, Fang Du, Jie Liu, Xiaoquan Li, Xu Zhao

**Affiliations:** ^1^ Emergency and Critical Care Center Renmin Hospital Hubei University of Medicine Shiyan China

**Keywords:** cancer, meta‐analysis, *PARP1*, risk, rs1136410 C>T

## Abstract

Poly(ADP‐ribose) polymerase‐1 (PARP‐1) is a nuclear chromatin‐associated enzyme involved in the DNA damage response. SNP rs1136410 C>T, the most studied polymorphism in *PARP‐1* gene, is highly implicated in the susceptibility of cancer. However, the roles of *PARP‐1* rs1136410 C>T on cancer risk vary from different studies. We comprehensively screened all qualified publications from several databases, including PubMed, EMBASE, MEDLINE, CNKI and Wanfang. The searching was updated to April 2020. Our meta‐analysis included 60 articles with 65 studies, comprised of a total of 23 996 cases with cancer and 33 015 controls. Overall, pooled data showed that the *PARP‐1* rs1136410 C>T polymorphism was significantly but a border‐line associated with an increased risk of overall cancer (CC vs. TT/TC: OR = 1.11, 95% CI = 1.00‐1.24; C vs T: OR = 1.07, 95% CI = 1.01‐1.14). Subgroup analysis indicated that rs1136410 C allele contributed to high risk among gastric, thyroid, and cervical cancer, but lower risk among brain cancer. Furthermore, increased cancer risk was detected in the subgroups of Asian, controls from population‐based design studies, and HWE ≤ 0.05 studies. Sensitivity analysis and Egger's test showed that results of the meta‐analysis were fairly stable. The current study indicated that *PARP1* rs1136410 C>T polymorphism may have an impact on certain types of cancer susceptibility.

## 1 INTRODUCTION

According to the Global Cancer Statistics 2018, about 18.1 million new cancer cases and 9.6 million cancer deaths occur worldwide in 2018.^1^ Single nucleotide polymorphisms (SNPs), the most frequent type of genetic alterations, have been proved to contribute to cancer susceptibility.^2^ Yet, all the identified SNPs only account for a small portion of cancer risk.

Poly(ADP‐ribose) polymerases (PARP), also known as ADP‐ribosyl‐transferase diphtheria toxin‐like (ARTDs), are evolutionary‐conserved family of proteins involved in diverse biological.^3^ In general, PARP‐1 senses the DNA damage and recruits critical repair proteins (eg XRCC1, DNA‐PK) to the damaged site.^4^, ^5^, ^6^ The human *PARP‐1* gene, located on chromosome 1q41‐42, spans about 47.3 kb and consists of 23 exons. Numerous SNPs have been identified in *PARP‐1* gene. Among them, rs1136410 T>C (Val762Ala) is a non‐synonymous polymorphism that could change valine to alanine. *PARP‐1* rs1136410 T>C genetic polymorphism was previously investigated in various types of cancer. However, the results of epidemiological studies are inconsistent and contradictory. The present work aims to fill this gap in the literature by presenting the latest updated meta‐analysis of the available evidence in elucidating the relationship of *PARP‐1* rs1136410 T>C and the risk of cancer.

## 2 MATERIALS AND METHODS

### 2.1 Publication search

PubMed, EMBASE, MEDLINE, Wanfang, and CNKI were searched for English/Chinese‐language articles published from January 1990, through April 2020. The following syntaxes were used: (a) PARP‐1 or poly (ADP‐ribose) polymerase 1 or PARP1 or ADPRT or ADPRT1 or rs1136410 C>T; (b) SNPs or polymorphisms or polymorphism or variants; (c) cancer or cancers or carcinoma or tumour or neoplasm. To obtain other appropriate publications, we also manually examined the references of the selected articles. Eligible criteria were as follows: (a) case‐control study; (b) assessing rs1136410 C>T and cancer risk; and (c) enough information in allele frequency. Editorials, reviews, meta‐analysis, case‐only studies, duplicate studies were ruled out.

### 2.2 Data extraction

For each included study, a reviewer (H. Li) abstracted relevant study characteristics: (ie authors name, publication year, cancer type, ethnicity of the study subject, control source, genotype method, score, allelic frequency) and classified the data into a structured form. Another reviewer (Y. Zha) checked all data for completeness and accuracy. Disagree parts (the conflicted information extraction by the two authors) were resolved through discussion until consensus was made.

### 2.3 Statistical methods

We used a chi‐square test to determine if genotype frequencies in controls of each study conformed to Hardy‐Weinberg equilibrium (HWE). *P* > .05 indicates not violating HWE. The association between *PARP‐1* rs1136410 C>T and cancer risk were assessed by calculating ORs with the corresponding 95% CIs. Significant heterogeneity exists if *I* ^2^ > 50%. If so, the random‐effect model was adopted, otherwise, the fixed‐effect model was used. Subgroup analyses by ethnicity, cancer type, source of control, and HWE in controls were performed to detect the source of heterogeneity. We also assessed the quality of each included study, the detailed method was described elsewhere.^7^ Sensitivity analysis was performed by re‐calculating the overall ORs when each study is removed at a time. Egger's regression test showing the funnel plot asymmetry was conducted to determine publication bias. STATA software version 11.0 (Stata Corporation, College Station, TX) was used for statistical analysis. All the statistics were two‐sides with *P* value < .05 implies a significant finding.

## 3 RESULTS

### 3.1 Study characteristics

Initial retrieval from PubMed, EMBASE, and MEDLINE databases got a total of 298 potentially relevant published records. We also obtained 12 articles from CNKI and Wanfang database. After titles and abstracts screening, 248 not relevant records were excluded. 4 articles were excluded due to being covered by others. A total of 58 case‐control design articles with reporting frequency of genotypes were included. Additionally, 2 eligible articles were also identified from references cited in the obtained articles. Ultimately, 65 studies from 60 publications were included (Table S1). A total of 23 996 cases with cancer and 33 015 controls were enrolled into this study for analysing, shown in Table S2 and Figure S1.

### 3.2 Quantitative analysis

**FIGURE 1:**
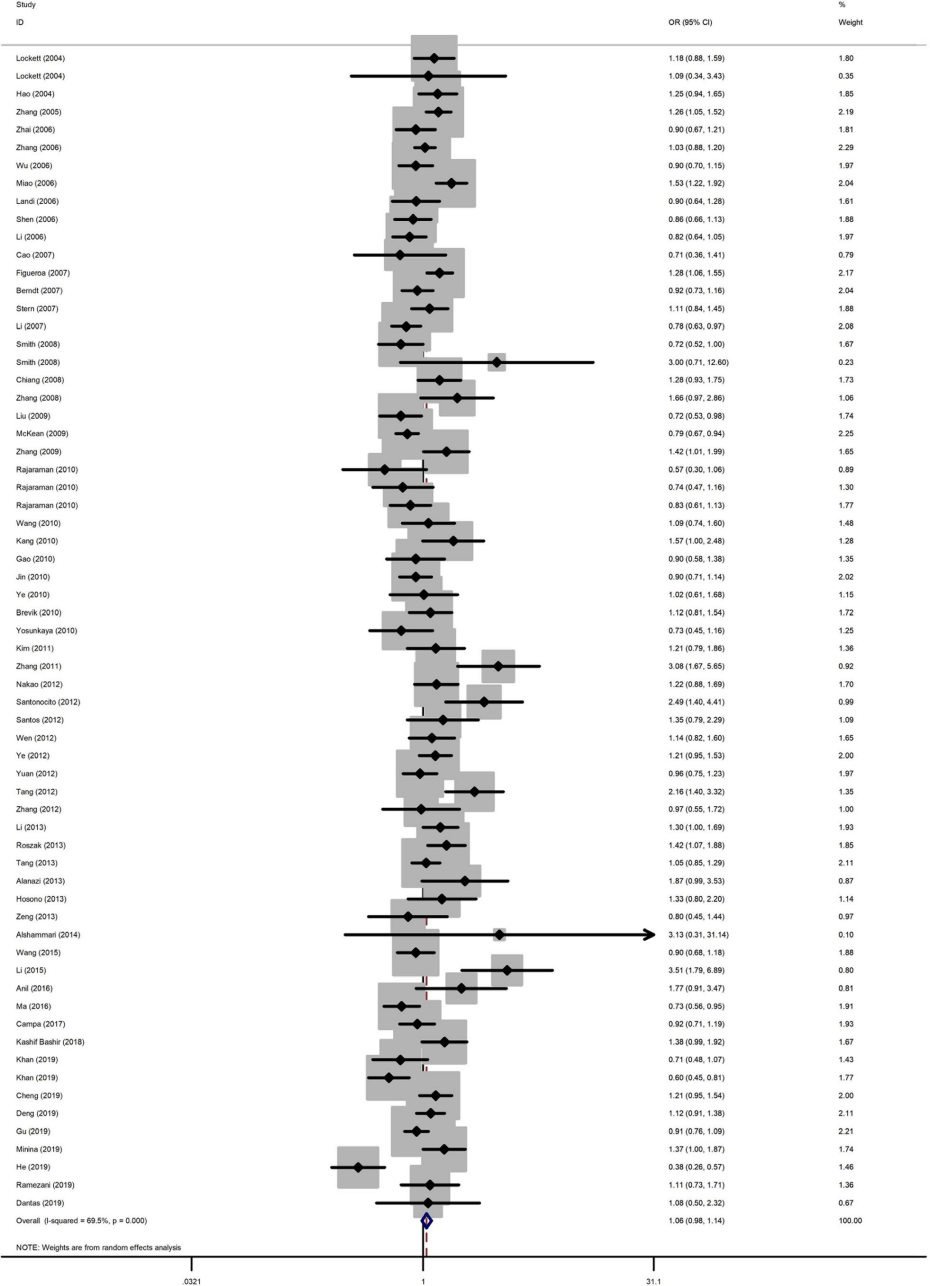
Forest plot of studies evaluating pooled ORs of cancer risk under the dominant comparison model. Squares represent risk estimates of each study. The horizontal line represents study specific ORs and 95% CIs. The diamonds represent pooled estimates and 95%

The main results of the meta‐analysis were shown in Table 1 and Figure 1. The pooled results indicated that rs1136410 C>T was associated with overall cancer risk in the recessive model (CC vs. TT/TC: OR = 1.11, 95% CI = 1.00‐1.24) and allele model (C vs. T: OR = 1.07, 95% CI = 1.01‐1.14), but not in other models. However, we observed that polymorphism rs1136410 C>T could confer to increased risk in gastric cancer; thyroid cancer; cervical cancer, whereas it is associated with decreased risk of brain cancer. Subgroup analysis by ethnicity showed that rs1136410 C allele had a contributing effect on cancer in Asian. In Caucasian and African, no significant association was detected. In terms of source of controls, population‐based controls were associated with the increased risk of cancer. Further subgroup analysis by HWE in controls revealed that rs1136410 C>T could not impact cancer risk in studies of HWE > 0.05, whereas studies of HWE ≤ 0.05 could impact cancer risk.

**Table 1:** Meta‐analysis of *PARP‐1* rs1136410 polymorphism

Variables	Homozygous		Heterozygous		Recessive		Dominant		Allele	
	CC vs TT		TC vs TT		CC vs TT/TC		TC/CC vs TT		C vs T	
	OR (95% CI)	P ^het^	OR (95% CI)	P ^het^	OR (95% CI)	P ^het^	OR (95% CI)	P ^het^	OR (95% CI)	P ^het^
All	1.20 (0.99‐1.20)	<0.001	1.03 (0.96‐1.10)	<0.001	**1.11 (1.00‐1.24)**	<0.001	1.06 (0.98‐1.14)	<0.001	**1.07 (1.01‐1.14)**	<0.001
Cancer type										
Prostate	1.06 (0.49‐2.28)	0.022	0.97 (0.84‐1.13)	0.877	1.07 (0.51‐2.23)	0.027	0.97 (0.84‐1.12)	0.496	0.99 (0.83‐1.21)	0.167
Others	0.93 (0.73‐1.17)	0.144	0.97 (0.85‐1.10)	0.298	0.92 (0.78‐1.09)	0.367	0.97 (0.83‐1.13)	0.076	0.97 (0.85‐1.10)	0.023
Lung	0.88 (0.44‐1.73)	<0.001	0.93 (0.69‐1.25)	0.001	0.94 (0.57‐1.54)	<0.001	0.90 (0.62‐1.31)	<0.001	0.93 (0.68‐1.28)	<0.001
Breast	0.85 (0.65‐1.11)	0.104	0.97 (0.84‐1.12)	0.106	0.87 (0.70‐1.08)	0.209	0.95 (0.81‐1.12)	0.035	0.95 (0.83‐1.09)	0.015
Bladder	0.99 (0.70‐1.40)	0.850	1.10 (0.84‐1.44)	0.057	0.96 (0.70‐1.34)	0.818	1.09 (0.86‐1.39)	0.083	1.07 (0.90‐1.26)	0.159
Gastric	**1.77 (1.24‐2.52)**	0.016	**1.36 (1.18‐1.57)**	0.742	**1.54 (1.08‐2.20)**	0.005	**1.46 (1.28‐1.66)**	0.425	**1.38 (1.17‐1.62)**	0.016
Melanoma	2.21 (0.43‐11.43)	0.160	1.69 (0.69‐4.15)	<0.001	1.80 (0.49‐6.70)	0.243	1.79 (0.70‐4.57)	<0.001	1.78 (0.74‐4.24)	<0.001
Colorectal	1.17 (0.80‐1.73)	0.067	1.08 (0.94‐1.24)	0.827	1.13 (0.81‐1.58)	0.102	1.09 (0.96‐1.24)	0.452	1.09 (0.93‐1.28)	0.073
Thyroid	**1.44 (1.10‐1.88)**	0.935	1.24 (0.99‐1.57)	0.947	**1.29 (1.04‐1.61)**	0.843	**1.33 (1.08‐1.64)**	0.949	**1.26 (1.10‐1.45)**	0.817
Brain	1.01 (0.86‐1.19)	0.359	**0.74 (0.60‐0.92)**	<0.001	1.14 (0.92‐1.42)	0.040	**0.82 (0.69‐0.96)**	0.003	0.93 (0.84‐1.04)	0.022
Pancreatic	1.80 (0.73‐4.56)	0.005	1.26 (0.81‐1.97)	0.028	1.53 (0.73‐3.23)	0.018	1.44 (0.82‐2.54)	0.001	1.41 (0.85‐2.35)	<0.001
Cervical	1.68 (0.91‐3.10)	0.036	1.14 (0.91‐1.43)	0.252	1.59 (0.82‐3.07)	0.011	**1.26 (1.06‐1.50)**	0.444	**1.28 (1.08‐1.52)**	0.201
Ethnicity										
Caucasian	0.96 (0.79‐1.16)	0.090	0.97 (0.88‐1.08)	<0.001	0.98 (0.81‐1.19)	0.092	0.98 (0.88‐1.09)	<0.001	0.99 (0.90‐1.09)	<0.001
African	–	–	1.64 (0.62‐4.37)	0.279	–	–	1.64 (0.62‐4.37)	0.279	1.60 (0.63‐4.10)	0.287
Asian	**1.20 (1.02‐1.40)**	<0.001	1.07 (0.97‐1.18)	<0.001	**1.17 (1.03‐1.33)**	<0.001	**1.12 (1.01‐1.23)**	<0.001	**1.12 (1.04‐1.21)**	<0.001
Source of control										
HB	1.61 (0.90‐2.89)	<0.001	0.99 (0.91‐1.07)	<0.001	1.11 (0.98‐1.26)	<0.001	1.02 (0.94‐1.10)	<0.001	1.04 (0.97‐1.11)	<0.001
PB	**2.61 (1.39‐4.91)**	0.010	**1.18 (1.01‐1.38)**	0.001	1.10 (0.87‐1.39)	0.049	**1.22 (1.03‐1.44)**	<0.001	**1.20 (1.03‐1.40)**	<0.001
FB	1.22 (0.54‐2.78)	–	1.10 (0.80‐1.54)	0.001	1.18 (0.52‐2.66)	–	1.12 (0.81‐1.54)	–	1.11 (0.84‐1.45)	–
Score										
≤9	1.15 (0.94‐1.42)	<0.001	1.04 (0.91‐1.18)	<0.001	1.17 (0.98‐1.39)	<0.001	1.08 (0.95‐1.23)	<0.001	1.11 (0.997‐1.23)	<0.001
>9	1.10 (0.94‐1.30)	<0.001	1.03 (0.96‐1.11)	0.001	1.08 (0.94‐1.24)	<0.001	1.05 (0.97‐1.14)	<0.001	1.05 (0.97‐1.12)	<0.001
HWE										
>0.05	1.03 (0.91‐1.17)	<0.001	1.004 (0.93‐1.08)	<0.001	1.05 (0.95‐1.16)	<0.001	1.02 (0.95‐1.11)	<0.001	1.03 (0.97‐1.10)	<0.001
≤0.05	**1.59 (1.12‐2.26)**	<0.001	1.17 (0.97‐1.42)	0.007	**1.47 (1.06‐2.05)**	<0.001	**1.27 (1.03‐1.56)**	<0.001	**1.27 (1.06‐1.51)**	<0.001

Abbreviations: FB, family based; HB, hospital based; Het, heterogeneity; PB, population based.

Bold values are significance.

### 3.3 Heterogeneity and sensitivity analysis

The *Q* test (*P* < .001) implied an existence of significant heterogeneity under all the genetic models. Therefore, a random‐effect model was applied to produce ORs and 95% CIs. In addition, the sequential sensitivity analysis was conducted to give an evaluation of the impact of a single study on the pooled ORs. The data of meta‐analysis is of great reliability, as no statistical fluctuation of the pooled ORs incurred after omitting in each study (Figure S2).

### 3.4 Publication bias

Begg's funnel plot and quantitative Egger's test were adopted to test the publication bias of the current meta‐analysis. The outlines of the Begg's funnel plots were rather symmetric, indicating the absence of any significant publication bias (Figure S3). Statistical evidence of Egger's test also confirms a none‐existence of publication bias among the studies.

## 4 DISCUSSION

The findings of this study suggest that *PARP‐1* rs1136410 C>T polymorphism has a board‐line significant relationship with overall cancer risk. However, further stratified analyses revealed that this polymorphism could predispose to gastric cancer, thyroid cancer, cervical cancer, but protects from brain cancer.

Due to the critical roles of PARP‐1 protein in cancer, the possible role of *PARP‐1* gene SNPs in cancer susceptibility has evoked intensive investigation. The latest meta‐analysis regarding this topic was conducted by Hua et al in 2013.^8^ Null significant association between *PARP‐1* rs1136410 C>T and overall cancer risk was detected in their analysis. Our meta‐analysis is the most updated and comprehensive study thus far to investigate the relationship between *PARP‐1* gene rs1136410 C>T variant and predisposition to cancer. The rs1136410 C>T variant failed to predispose to overall cancer risk. This result hinted a clue that rs1136410 C>T variant itself may not be strong enough to impact the carcinogenesis or its effect is modified by other factors. Subjects with rs1136410 C allele were more susceptible to gastric cancer, thyroid cancer, cervical cancer, but less susceptible to brain cancer. Such a phenomenon may be attributed to the tissue‐specific expression levels of PARP‐1. HWE is the principal law in population genetic studies. The selected samples are representative if the analysed SNPs conform with HWE. 11 included studies violated HWE, and the rest 54 studies conformed with HWE. The pooled results of HWE>0.05 failed to provide a significant relationship, we believe these results were representative.

Compared to the previous meta‐analyses,^8^, ^9^ the conclusion here is more credible as including a remarkably larger number of studies (65 studies vs. 21 studies and 28 studies). Moreover, sensitivity analysis and the none‐existence of publication bias indicated the strength of the conclusion. Nevertheless, we also recognized the limitations. First, significant heterogeneity exists across studies. Thus, the interpretation of conclusion should be cautious. Second, the calculated relationship was only based on unadjusted estimates. Elucidating an explicit link between *PARP‐1* gene rs1136410 C>T and cancer risk no doubt requires a well‐design study with phenotypically homogeneous subjects as well as the inclusion of meticulous analyses of gene‐gene and gene‐environment interactions. Third, genotyping bias was inevitable as several different genotyping methods were adopted across studies. Last, the generalization of the findings is limited as most of the study populations were of Asians and Caucasians, and thus additional studies in other ethnic groups are warranted.

## 5 CONCLUSION

In conclusion, our meta‐analysis showed that *PARP‐1* gene rs1136410 C>T polymorphism may contribute to increased cancer risk among Asian populations. More investigations are encouraged to provide more evidence regarding the role of *PARP‐1* rs1136410 C>T polymorphism to the aetiology of cancer predisposition.

## CONFLICT OF INTEREST

The authors declare no competing financial interests.

## AUTHOR CONTRIBUTION

**Hu‐Nian Li:** Resources (equal); Writing‐original draft (equal). **Yong‐Jiu Zha:** Data curation (equal); Writing‐original draft (equal). **Jie Liu:** Methodology (equal); Software (supporting); Validation (equal). **Fang Du:** Investigation (equal); Software (equal); Supervision (equal). **Xiao‐Quan Li:** Supervision (equal); Visualization (equal); Writing‐review & editing (equal). **Xu Zhao:** Funding acquisition (equal); Resources (equal); Supervision (equal); Visualization (equal); Writing‐review & editing (equal).

## Supporting information

App S1

## Data Availability

All the data were available upon request.
