# Supplementary material for: Contributions of PARP‐1 rs1136410 C>T polymorphism to the development of cancer
Source: J Cell Mol Med. 2020 Oct 27;24(24):14639–44. doi: 10.1111/jcmm.16027 (PMC7753995; doi:10.1111/jcmm.16027)
Supplement: Supplementary file 1 — App S1 [file JCMM-24-14639-s001.doc]

**Table S1.** Included case-control studies analyzed

| **No.** | **Reference** |
| --- | --- |
| 1. | **Lockett KL, Hall MC, Xu J, Zheng SL, Berwick M, Chuang SC, Clark PE, Cramer SD, Lohman K, Hu JJ.** The ADPRT V762A genetic variant contributes to prostate cancer susceptibility and deficient enzyme function. *Cancer Res*. 2004; 64: 6344-8. |
| 2. | **Berndt SI, Huang WY, Fallin MD, Helzlsouer KJ, Platz EA, Weissfeld JL, Church TR, Welch R, Chanock SJ, Hayes RB.** Genetic variation in base excision repair genes and the prevalence of advanced colorectal adenoma. *Cancer Res*. 2007; 67: 1395-404. |
| 3. | **Brevik A, Joshi AD, Corral R, Onland-Moret NC, Siegmund KD, Le Marchand L, Baron JA, Martinez ME, Haile RW, Ahnen DJ, Sandler RS, Lance P, Stern MC.** Polymorphisms in base excision repair genes as colorectal cancer risk factors and modifiers of the effect of diets high in red meat. *Cancer Epidemiol Biomarkers Prev*. 2010; 19: 3167-73. |
| 4. | **Cao WH, Wang X, Frappart L, Rigal D, Wang ZQ, Shen Y, Tong WM.** Analysis of genetic variants of the poly(ADP-ribose) polymerase-1 gene in breast cancer in French patients. *Mutat Res*. 2007; 632: 20-8. |
| 5. | **Chiang FY, Wu CW, Hsiao PJ, Kuo WR, Lee KW, Lin JC, Liao YC, Juo SH.** Association between polymorphisms in DNA base excision repair genes XRCC1, APE1, and ADPRT and differentiated thyroid carcinoma. *Clin Cancer Res*. 2008; 14: 5919-24. |
| 6. | **Figueroa JD, Malats N, Real FX, Silverman D, Kogevinas M, Chanock S, Welch R, Dosemeci M, Tardon A, Serra C, Carrato A, Garcia-Closas R, Castano-Vinyals G, Rothman N, Garcia-Closas M.** Genetic variation in the base excision repair pathway and bladder cancer risk. *Hum Genet*. 2007; 121: 233-42. |
| 7. | **Gao R, Price DK, Dahut WL, Reed E, Figg WD.** Genetic polymorphisms in XRCC1 associated with radiation therapy in prostate cancer. *Cancer Biol Ther*. 2010; 10: 13-8. |
| 8. | **Hao B, Wang H, Zhou K, Li Y, Chen X, Zhou G, Zhu Y, Miao X, Tan W, Wei Q, Lin D, He F.** Identification of genetic variants in base excision repair pathway and their associations with risk of esophageal squamous cell carcinoma. *Cancer Res*. 2004; 64: 4378-84. |
| 9. | **Jin XM, Kim HN, Lee IK, Park KS, Kim HJ, Choi JS, Juhng SW, Choi C.** PARP-1 Val762Ala polymorphism is associated with reduced risk of non-Hodgkin lymphoma in Korean males. *BMC Med Genet*. 2010; 11: 38. |
| 10. | **Kang SL LY, He WT, Liu T, Li X.** Association between PARP-1 polymorphisms and susceptibility to gastric cancer. *World J Gastroenterol*. 2010; 18: 1434-41. |
| 11. | **Kim J, Pyun JA, Cho SW, Lee K, Kwack K.** Lymph node metastasis of gastric cancer is associated with the interaction between poly (ADP-ribose) polymerase 1 and matrix metallopeptidase 2. *DNA Cell Biol*. 2011; 30: 1011-7. |
| 12. | **Landi S, Gemignani F, Canzian F, Gaborieau V, Barale R, Landi D, Szeszenia-Dabrowska N, Zaridze D, Lissowska J, Rudnai P, Fabianova E, Mates D, Foretova L, Janout V, Bencko V, Gioia-Patricola L, Hall J, Boffetta P, Hung RJ, Brennan P.** DNA repair and cell cycle control genes and the risk of young-onset lung cancer. *Cancer Res*. 2006; 66: 11062-9. |
| 13. | **Li C, Hu Z, Lu J, Liu Z, Wang LE, El-Naggar AK, Sturgis EM, Spitz MR, Wei Q.** Genetic polymorphisms in DNA base-excision repair genes ADPRT, XRCC1, and APE1 and the risk of squamous cell carcinoma of the head and neck. *Cancer*. 2007; 110: 867-75. |
| 14. | **Li C, Liu Z, Wang LE, Strom SS, Lee JE, Gershenwald JE, Ross MI, Mansfield PF, Cormier JN, Prieto VG, Duvic M, Grimm EA, Wei Q.** Genetic variants of the ADPRT, XRCC1 and APE1 genes and risk of cutaneous melanoma. *Carcinogenesis*. 2006; 27: 1894-901. |
| 15. | **Li LM, Zeng XY, Ji L, Fan XJ, Li YQ, Hu XH, Qiu XQ, Yu HP.** [Association of XPC and XPG polymorphisms with the risk of hepatocellular carcinoma]. *Zhonghua Gan Zang Bing Za Zhi*. 2010; 18: 271-5. |
| 16. | **Li Y, Li S, Wu Z, Hu F, Zhu L, Zhao X, Cui B, Dong X, Tian S, Wang F, Zhao Y.** Polymorphisms in genes of APE1, PARP1, and XRCC1: risk and prognosis of colorectal cancer in a northeast Chinese population. *Med Oncol*. 2013; 30: 505. |
| 17. | **Liu Y, Scheurer ME, El-Zein R, Cao Y, Do KA, Gilbert M, Aldape KD, Wei Q, Etzel C, Bondy ML.** Association and interactions between DNA repair gene polymorphisms and adult glioma. *Cancer Epidemiol Biomarkers Prev*. 2009; 18: 204-14. |
| 18. | **McKean-Cowdin R, Barnholtz-Sloan J, Inskip PD, Ruder AM, Butler M, Rajaraman P, Razavi P, Patoka J, Wiencke JK, Bondy ML, Wrensch M.** Associations between polymorphisms in DNA repair genes and glioblastoma. *Cancer Epidemiol Biomarkers Prev*. 2009; 18: 1118-26. |
| 19. | **Miao X, Zhang X, Zhang L, Guo Y, Hao B, Tan W, He F, Lin D.** Adenosine diphosphate ribosyl transferase and x-ray repair cross-complementing 1 polymorphisms in gastric cardia cancer. *Gastroenterology*. 2006; 131: 420-7. |
| 20. | **Nakao M, Hosono S, Ito H, Watanabe M, Mizuno N, Sato S, Yatabe Y, Yamao K, Ueda R, Tajima K, Tanaka H, Matsuo K.** Selected polymorphisms of base excision repair genes and pancreatic cancer risk in Japanese. *J Epidemiol*. 2012; 22: 477-83. |
| 21. | **Rajaraman P, Hutchinson A, Wichner S, Black PM, Fine HA, Loeffler JS, Selker RG, Shapiro WR, Rothman N, Linet MS, Inskip PD.** DNA repair gene polymorphisms and risk of adult meningioma, glioma, and acoustic neuroma. *Neuro Oncol*. 2010; 12: 37-48. |
| 22. | **Santonocito C, Scapaticci M, Penitente R, Paradisi A, Capizzi R, Lanza-Silveri S, Ficarra S, Landi F, Zuppi C, Capoluongo E.** Polymorphisms in base excision DNA repair genes and association with melanoma risk in a pilot study on Central-South Italian population. *Clin Chim Acta*. 2012; 413: 1519-24. |
| 23. | **Santos LS, Branco SC, Silva SN, Azevedo AP, Gil OM, Manita I, Ferreira TC, Limbert E, Rueff J, Gaspar JF.** Polymorphisms in base excision repair genes and thyroid cancer risk. *Oncol Rep*. 2012; 28: 1859-68. |
| 24. | **Shen M, Zheng T, Lan Q, Zhang Y, Zahm SH, Wang SS, Holford TR, Leaderer B, Yeager M, Welch R, Kang D, Boyle P, Zhang B, Zou K, Zhu Y, Chanock S, Rothman N.** Polymorphisms in DNA repair genes and risk of non-Hodgkin lymphoma among women in Connecticut. *Hum Genet*. 2006; 119: 659-68. |
| 25. | **Smith TR, Levine EA, Freimanis RI, Akman SA, Allen GO, Hoang KN, Liu-Mares W, Hu JJ.** Polygenic model of DNA repair genetic polymorphisms in human breast cancer risk. *Carcinogenesis*. 2008; 29: 2132-8. |
| 26. | **Stern MC, Conti DV, Siegmund KD, Corral R, Yuan JM, Koh WP, Yu MC.** DNA repair single-nucleotide polymorphisms in colorectal cancer and their role as modifiers of the effect of cigarette smoking and alcohol in the Singapore Chinese Health Study. *Cancer Epidemiol Biomarkers Prev*. 2007; 16: 2363-72. |
| 27. | **Tang LY, Chen LJ, Qi ML, Su Y, Su FX, Lin Y, Wang KP, Jia WH, Zhuang ZX, Ren ZF.** Effects of passive smoking on breast cancer risk in pre/post-menopausal women as modified by polymorphisms of PARP1 and ESR1. *Gene*. 2013; 524: 84-9. |
| 28. | **Tang X HZ, Li Y, Jiang X** Correlation between the nucleotide polymorphisms of PARP-1 and the susceptibility of gastric cancer in Hui Ethnic group of Linxia Hui autonomous prefecture. *Journal of Medical Research*. 2012; 41: 151-4. |
| 29. | **Wang M, Qin C, Zhu J, Yuan L, Fu G, Zhang Z, Yin C.** Genetic variants of XRCC1, APE1, and ADPRT genes and risk of bladder cancer. *DNA Cell Biol*. 2010; 29: 303-11. |
| 30. | **Wen YY, Pan XF, Loh M, Tian Z, Yang SJ, Lv SH, Huang WZ, Huang H, Xie Y, Soong R, Yang CX.** ADPRT Val762Ala and XRCC1 Arg194Trp polymorphisms and risk of gastric cancer in Sichuan of China. *Asian Pac J Cancer Prev*. 2012; 13: 2139-44. |
| 31. | **Wu X, Gu J, Grossman HB, Amos CI, Etzel C, Huang M, Zhang Q, Millikan RE, Lerner S, Dinney CP, Spitz MR.** Bladder cancer predisposition: a multigenic approach to DNA-repair and cell-cycle-control genes. *Am J Hum Genet*. 2006; 78: 464-79. |
| 32. | **Ye CC HZ, Zhou CY.** APE1 D148E, PARP1 V762A and XRCC1 R399Q polymorphisms and genetic susceptibility to colorectal cancer. *World J Gastroenterol* 2010; 18: 1275-9. |
| 33. | **Ye F, Cheng Q, Hu Y, Zhang J, Chen H.** PARP-1 Val762Ala polymorphism is associated with risk of cervical carcinoma. *PLoS One*. 2012; 7: e37446. |
| 34. | **Yosunkaya E, Kucukyuruk B, Onaran I, Gurel CB, Uzan M, Kanigur-Sultuybek G.** Glioma risk associates with polymorphisms of DNA repair genes, XRCC1 and PARP1. *Br J Neurosurg*. 2010; 24: 561-5. |
| 35. | **Yuan H, Li H, Ma H, Niu Y, Wu Y, Zhang S, Hu Z, Shen H, Chen N.** Genetic polymorphisms in key DNA repair genes and risk of head and neck cancer in a Chinese population. *Exp Ther Med*. 2012; 3: 719-24. |
| 36. | **Zhai X, Liu J, Hu Z, Wang S, Qing J, Wang X, Jin G, Gao J, Wang X, Shen H.** Polymorphisms of ADPRT Val762Ala and XRCC1 Arg399Glu and risk of breast cancer in Chinese women: a case control analysis. *Oncol Rep*. 2006; 15: 247-52. |
| 37. | **Zhang L, Ruan Z, Hong Q, Gong X, Hu Z, Huang Y, Xu A.** Single nucleotide polymorphisms in DNA repair genes and risk of cervical cancer: A case-control study. *Oncol Lett*. 2012; 3: 351-62. |
| 38. | **Zhang M, Qureshi AA, Guo Q, Han J.** Genetic variation in DNA repair pathway genes and melanoma risk. *DNA Repair (Amst)*. 2011; 10: 111-6. |
| 39. | **Zhang Q, Li Y, Li X, Zhou W, Shi B, Chen H, Yuan W.** PARP-1 Val762Ala polymorphism, CagA+ H. pylori infection and risk for gastric cancer in Han Chinese population. *Mol Biol Rep*. 2009; 36: 1461-7. |
| 40. | **Zhang Q LY, Li X, ZhouW, Shi B.** Polymorphisms Val762Ala in PARP-1 and gastric cancer. *Chin J Gene Surg*. 2008; 23: 706–9. |
| 41. | **Zhang X, Miao X, Liang G, Hao B, Wang Y, Tan W, Li Y, Guo Y, He F, Wei Q, Lin D.** Polymorphisms in DNA base excision repair genes ADPRT and XRCC1 and risk of lung cancer. *Cancer Res*. 2005; 65: 722-6. |
| 42. | **Zhang Y, Newcomb PA, Egan KM, Titus-Ernstoff L, Chanock S, Welch R, Brinton LA, Lissowska J, Bardin-Mikolajczak A, Peplonska B, Szeszenia-Dabrowska N, Zatonski W, Garcia-Closas M.** Genetic polymorphisms in base-excision repair pathway genes and risk of breast cancer. *Cancer Epidemiol Biomarkers Prev*. 2006; 15: 353-8. |
| 43. | **Alanazi M, Pathan AA, Abduljaleel Z, Shaik JP, Alabdulkarim HA, Semlali A, Bazzi MD, Parine NR.** Association between PARP-1 V762A polymorphism and breast cancer susceptibility in Saudi population. *PLoS One*. 2013; 8: e85541. |
| 44. | **Alshammari AH, Shalaby MA, Alanazi MS, Saeed HM.** Novel mutations of the PARP-1 gene associated with colorectal cancer in the Saudi population. *Asian Pac J Cancer Prev*. 2014; 15: 3667-73. |
| 45. | **Anil S, Gopikrishnan PB, Basheer AB, Vidyullatha BG, Alogaibi YA, Chalisserry EP, Javed F, Dalati MH, Vellappally S, Hashem MI, Divakar DD.** Association of Poly (ADP-Ribose) Polymerase 1 Variants with Oral Squamous Cell Carcinoma Susceptibility in a South Indian Population. *Asian Pac J Cancer Prev*. 2016; 17: 4107-11. |
| 46. | **Bashir K, Sarwar R, Saeed S, Mahjabeen I, Kayani MA.** Interaction among susceptibility genotypes of PARP1 SNPs in thyroid carcinoma. *PLoS One*. 2018; 13: e0199007. |
| 47. | **Campa D, Obazee O, Pastore M, Panzuto F, Lico V, Greenhalf W, Katzke V, Tavano F, Costello E, Corbo V, Talar-Wojnarowska R, Strobel O, Zambon CF, Neoptolemos JP, Zerboni G, Kaaks R, Key TJ, Lombardo C, Jamroziak K, Gioffreda D, Hackert T, Khaw KT, Landi S, Milanetto AC, Landoni L, Lawlor RT, Bambi F, Pirozzi F, Basso D, Pasquali C, Capurso G, Canzian F.** Lack of Association for Reported Endocrine Pancreatic Cancer Risk Loci in the PANDoRA Consortium. *Cancer Epidemiol Biomarkers Prev*. 2017; 26: 1349-51. |
| 48. | **Cheng J, Zhuo Z, Zhao P, Zhu J, Xin Y, Zhang J, Li P, Gao Y, He J, Zheng B.** PARP1 gene polymorphisms and neuroblastoma susceptibility in Chinese children. *J Cancer*. 2019; 10: 4159-64. |
| 49. | **Dantas RN, Souza AM, Herrero SST, Kassab P, Malheiros CA, Lima EM.** Association between PSCA, TNF-alpha, PARP1 and TP53 Gene Polymorphisms and Gastric Cancer Susceptibility in the Brazilian Population. *Asian Pac J Cancer Prev*. 2020; 21: 43-8. |
| 50. | **Deng Y, Zhou L, Li N, Wang M, Yao L, Dong S, Zhang M, Yang P, Hao Q, Wu Y, Lyu L, Jin T, Dai Z, Kang H.** Impact of four lncRNA polymorphisms (rs2151280, rs7763881, rs1136410, and rs3787016) on glioma risk and prognosis: A case-control study. *Mol Carcinog*. 2019; 58: 2218-29. |
| 51. | **Gu CY, Jin SM, Qin XJ, Zhu Y, Bo D, Lin GW, Shi GH, Ye DW.** Genetic variants in RTEL1 influencing telomere length are associated with prostate cancer risk. *J Cancer*. 2019; 10: 6170-4. |
| 52. | **Hosono S, Matsuo K, Ito H, Oze I, Hirose K, Watanabe M, Nakanishi T, Tajima K, Tanaka H.** Polymorphisms in base excision repair genes are associated with endometrial cancer risk among postmenopausal Japanese women. *Int J Gynecol Cancer*. 2013; 23: 1561-8. |
| 53. | **Khan AU, Mahjabeen I, Malik MA, Hussain MZ, Khan S, Kayani MA.** Modulation of brain tumor risk by genetic SNPs in PARP1gene: Hospital based case control study. *PLoS One*. 2019; 14: e0223882. |
| 54. | **Minina VI, Bakanova ML, Soboleva OA, Ryzhkova AV, Titov RA, Savchenko YA, Sinitsky MY, Voronina EN, Titov VA, Glushkov AN.** Polymorphisms in DNA repair genes in lung cancer patients living in a coal-mining region. *Eur J Cancer Prev*. 2019; 28: 522-8. |
| 55. | **Ramezani S, Sharafshah A, Mirzanejad L, Hadavi M.** Association of PARP1 rs4653734, rs907187 and rs1136410 variants with breast cancer risk among Iranian women. *Gene*. 2019; 712: 143954. |
| 56. | **Wang X, Ma KW, Zhao YG, Wang GJ, Li W.** XRCC1 rs25487 polymorphism is associated with lung cancer risk in epidemiologically susceptible Chinese people. *Genet Mol Res*. 2015; 14: 15530-8. |
| 57. | **Xiao-Bin Ma X-JW, Meng Wang, Zhi-Ming Dai, Tian-Bo Jin, Xing-Han Liu, Hua-Feng Kang, Shuai Lin, Peng Xu, Zhi-Jun Dai** Impact of the PARP1 rs1136410 and rs3219145 polymorphisms on susceptibility and clinicopathologic features of breast cancer in a Chinese population. *Transl Cancer Res*. 2016; 5: 520-8. |
| 58. | **Xun L, Qiyu Z, Jun Y, Wenting H, Wenche Z, Lei Z, Wenbo M, Zhongtian B, Kexiang Z, Xiaoliang Z.** The association between PARP-1 Val762Ala polymorphisms and its susceptibility to pancreatic cancer in Chinese Han population in west area. *Med J West China*. 2015; 27: 490-2. |
| 59. | **Yan HWJHHSCJTHPDDXW.** Correlation Analysis of ADPRT rs1136410 Polymorphism with the Occurrence of Non-small Cell Lung Cancer in Han Nationality from Northern Jiangsu. *China Pharmacy*. 2019; 30: 2258-62. |
| 60. | **Zeng L, You G, Tanaka H, Srivatanakul P, Ohta E, Viwatthanasittiphong C, Matharit M, Chenvidhya D, Jedpiyawongse A, Tanaka M, Fujii T, Sripa B, Ohshima K, Miwa M, Honjo S.** Combined effects of polymorphisms of DNA-repair protein genes and metabolic enzyme genes on the risk of cholangiocarcinoma. *Jpn J Clin Oncol*. 2013; 43: 1190-4. |

**Table S2. Main characteristics of included studies in the meta-analysis**

| Surname | Year | Cancer type | Country | Ethnicity | Control Source | Genotype method | Score | Case | | | | Control | | | | HWE |
| --- | --- | --- | --- | --- | --- | --- | --- | --- | --- | --- | --- | --- | --- | --- | --- | --- |
|  |  |  |  |  |  |  |  | TT | TC | CC | All | TT | TC | CC | All |  |
| Lockett | 2004 | Prostate | USA | Caucasian | HB | MassARRAY | 9 | 306 | 113 | 19 | 438 | 313 | 107 | 7 | 427 | 0.532 |
| Lockett | 2004 | Prostate | USA | African | HB | MassARRAY | 8 | 45 | 5 | 0 | 50 | 88 | 9 | 0 | 97 | 0.632 |
| Hao | 2004 | Esophageal | China | Asian | HB | PCR-RFLP | 8 | 125 | 212 | 77 | 414 | 168 | 230 | 81 | 479 | 0.880 |
| Zhang | 2005 | Lung | China | Asian | HB | PCR-RFLP | 10 | 307 | 509 | 184 | 1000 | 359 | 504 | 137 | 1000 | 0.057 |
| Zhai | 2006 | Breast | China | Asian | HB | PCR-RFLP | 9 | 100 | 153 | 49 | 302 | 197 | 331 | 111 | 639 | 0.164 |
| Zhang | 2006 | Breast | USA | Caucasian | PB | TaqMan | 11 | 1194 | 468 | 54 | 1716 | 963 | 361 | 47 | 1371 | 0.071 |
| Wu | 2006 | Bladder | USA | Caucasian | HB | TaqMan | 10 | 437 | 155 | 14 | 606 | 416 | 165 | 14 | 595 | 0.618 |
| Miao | 2006 | Gastric | China | Asian | HB | PCR-RFLP | 10 | 150 | 257 | 93 | 500 | 396 | 492 | 112 | 1000 | 0.026 |
| Landi | 2006 | Lung | Europe | Caucasian | HB | APEX | 6 | 207 | 75 | 10 | 292 | 211 | 84 | 12 | 307 | 0.325 |
| Shen | 2006 | NHL | USA | Caucasian | PB | TaqMan | 10 | 323 | 125 | 7 | 455 | 363 | 160 | 12 | 535 | 0.246 |
| Li | 2006 | Melanoma | USA | Caucasian | HB | PCR-RFLP | 10 | 437 | 147 | 18 | 602 | 413 | 173 | 17 | 603 | 0.827 |
| Cao | 2007 | Breast | France | Caucasian | HB | Sequence | 9 | 65 | 17 | 1 | 83 | 72 | 28 | 0 | 100 | 0.104 |
| Figueroa | 2007 | Bladder | Spain | Caucasian | HB | TaqMan | 11 | 825 | 294 | 19 | 1138 | 873 | 235 | 23 | 1131 | 0.130 |
| Berndt | 2007 | Colorectal | USA | Caucasian | PB | TaqMan | 10 | 492 | 179 | 20 | 691 | 488 | 183 | 31 | 702 | 0.012 |
| Stern | 2007 | Colorectal | Singapore | Asian | PB | TaqMan | 11 | 93 | 150 | 64 | 307 | 381 | 564 | 228 | 1173 | 0.457 |
| Li | 2007 | SCCHN | USA | Caucasian | HB | PCR-RFLP | 10 | 632 | 182 | 16 | 830 | 609 | 216 | 29 | 854 | 0.074 |
| Smith | 2008 | Breast | USA | Caucasian | HB | MassARRAY | 9 | 236 | 71 | 7 | 314 | 272 | 114 | 11 | 397 | 0.819 |
| Smith | 2008 | Breast | USA | African | HB | MassARRAY | 8 | 46 | 6 | 0 | 52 | 69 | 3 | 0 | 72 | 0.857 |
| Chiang | 2008 | Thyroid | China | Asian | HB | TaqMan | 6 | 86 | 139 | 58 | 283 | 168 | 221 | 80 | 469 | 0.616 |
| Zhang | 2008 | Gastric | China | Asian | HB | PCR-RFLP | 5 | 85 | 37 | 16 | 138 | 80 | 25 | 5 | 110 | 0.114 |
| Liu | 2009 | Glioma | USA | Caucasian | PB | MassARRAY | 6 | 267 | 95 | 10 | 372 | 236 | 117 | 12 | 365 | 0.587 |
| McKean | 2009 | Glioblastoma | USA | Caucasian | HB | MassARRAY | 11 | 713 | 251 | 23 | 987 | 1303 | 575 | 57 | 1935 | 0.501 |
| Zhang | 2009 | Gastric | China | Asian | HB | PCR-RFLP | 10 | 113 | 83 | 40 | 236 | 181 | 106 | 33 | 320 | 0.005 |
| Rajaraman | 2010 | Neuroma | USA | Caucasian | HB | TaqMan | 8 | 51 | 14 | 0 | 65 | 312 | 136 | 15 | 463 | 0.970 |
| Rajaraman | 2010 | Meningioma | USA | Caucasian | HB | TaqMan | 8 | 89 | 29 | 3 | 121 | 312 | 136 | 15 | 463 | 0.970 |
| Rajaraman | 2010 | Glioma | USA | Caucasian | HB | TaqMan | 8 | 242 | 91 | 7 | 340 | 312 | 136 | 16 | 464 | 0.804 |
| Wang | 2010 | Bladder | China | Asian | HB | PCR-RFLP | 5 | 68 | 120 | 46 | 234 | 78 | 127 | 48 | 253 | 0.771 |
| Kang | 2010 | Gastric | China | Asian | PB | SNaPshot | 5 | 70 | 67 | 13 | 150 | 88 | 50 | 14 | 152 | 0.089 |
| Gao | 2010 | Prostate | USA | Caucasian | HB | Sequence | 6 | 315 | 123 | 15 | 453 | 80 | 32 | 7 | 119 | 0.133 |
| Jin | 2010 | NHL | Korea | Asian | PB | PCR-HRM | 10 | 189 | 279 | 105 | 573 | 221 | 354 | 146 | 721 | 0.845 |
| Ye | 2010 | Colorectal | China | Asian | HB | MassARRAY | 8 | 40 |  |  | 122 | 52 |  |  | 157 |  |
| Brevik | 2010 | Colorectal | USA | Caucasian | FB | TaqMan | 8 | 196 | 100 | 12 | 308 | 239 | 110 | 12 | 361 | 0.880 |
| Yosunkaya | 2010 | Glioma | Turkey | Caucasian | HB | PCR-RFLP | 6 | 57 | 40 | 22 | 119 | 72 | 93 | 15 | 180 | 0.046 |
| Kim | 2011 | Gastric | Korea | Asian | HB | GoldenGate | 5 | 42 | 70 | 39 | 151 | 102 | 161 | 57 | 320 | 0.635 |
| Zhang | 2011 | Melanoma | USA | Caucasian | PB | Illumina | 6 | 169 | 43 | 1 | 213 | 189 | 16 | 0 | 205 | 0.561 |
| Nakao | 2012 | Pancreatic | Japanese | Asian | HB | TaqMan | 11 | 61 | 90 | 34 | 185 | 550 | 657 | 258 | 1465 | 0.012 |
| Santonocito | 2012 | Melanoma | Italy | Caucasian | PB | PCR | 8 | 100 | 59 | 8 | 167 | 78 | 21 | 0 | 99 | 0.238 |
| Santos | 2012 | Thyroid | Portugal | Caucasian | HB | TaqMan | 6 | 78 | 30 | 0 | 108 | 168 | 48 | 0 | 216 | 0.066 |
| Wen | 2012 | Gastric | China | Asian | HB | MassARRAY | 5 | 96 | 154 | 57 | 307 | 105 | 132 | 70 | 307 | 0.024 |
| Ye | 2012 | Cervical | China | Asian | HB | MA-PCR | 10 | 152 | 283 | 104 | 539 | 257 | 475 | 68 | 800 | <0.001 |
| Yuan | 2012 | HNC | China | Asian | HB | TaqMan | 8 | 138 | 193 | 64 | 395 | 300 | 431 | 152 | 883 | 0.895 |
| Tang | 2012 | Gastric | China | Asian | PB | PCR-RFLP | 5 | 122 | 56 | 22 | 200 | 162 | 40 | 8 | 210 | 0.011 |
| Zhang | 2012 | Cervical | China | Asian | HB | SNPstream | 4 | 25 | 39 | 16 | 80 | 54 | 83 | 39 | 176 | 0.508 |
| Li | 2013 | Colorectal | China | Asian | HB | PCR-RFLP | 8 | 134 | 228 | 89 | 451 | 222 | 319 | 85 | 626 | 0.078 |
| Roszak | 2013 | Cervical | Poland | Caucasian | PB | HRM | 10 | 295 | 129 | 22 | 446 | 361 | 114 | 16 | 491 | 0.066 |
| Tang | 2013 | Breast | China | Asian | HB | MassARRAY | 10 | 250 | 405 | 138 | 793 | 275 | 419 | 151 | 845 | 0.694 |
| Alanazi | 2013 | Breast | Saudi Arabia | Asian | PB | TaqMan | 9 | 65 | 27 | 7 | 99 | 75 | 20 | 1 | 96 | 0.793 |
| Hosono | 2013 | Endometrial | Japan | Asian | HB | TaqMan | 8 | 29 | 47 | 15 | 91 | 100 | 121 | 40 | 261 | 0.733 |
| Zeng | 2013 | Cholangiocarcinoma | Thailand | Asian | HB | PCR-RFLP | 8 | 40 | 11 | 43 | 94 | 35 | 11 | 48 | 94 | <0.001 |
| Alshammari | 2014 | Colorectal | Saudi Arabia | Asian | HB | Sequence | 8 | 47 | 2 | 1 | 50 | 49 | 1 | 0 | 50 | 0.943 |
| Wang | 2015 | Lung | China | Asian | HB | MassARRAY | 10 | 151 | 252 | 97 | 500 | 140 | 251 | 109 | 500 | 0.860 |
| Li | 2015 | Pancreatic | China | Asian | HB | MassARRAY | 6 | 21 | 24 | 21 | 66 | 54 | 24 | 9 | 87 | 0.021 |
| Anil | 2016 | OSCC | India | Asian | HB | TaqMan | 5 | 72 | 21 | 7 | 100 | 82 | 16 | 2 | 100 | 0.266 |
| Ma | 2016 | Breast | China | Asian | HB | MassARRAY | 10 | 182 | 201 | 75 | 458 | 162 | 227 | 110 | 499 | 0.073 |
| Campa | 2017 | Pancreatic | Italy | Caucasian | PB | KASPar | 11 | 246 | 78 | 9 | 333 | 1753 | 611 | 63 | 2427 | 0.266 |
| Bashir | 2018 | Thyroid | Pakistan | Asian | HB | ARMS-PCR | 10 | 82 | 97 | 276 | 455 | 93 | 91 | 216 | 400 | <0.001 |
| Khan | 2019 | Meningioma | Pakistan | Asian | HB | AS-PCR | 7 | 45 | 47 | 57 | 149 | 118 | 229 | 153 | 500 | 0.075 |
| Khan | 2019 | Glioma | Pakistan | Asian | HB | AS-PCR | 7 | 119 | 89 | 143 | 351 | 118 | 229 | 153 | 500 | 0.075 |
| Cheng | 2019 | Neuroblastoma | China | Asian | HB | TaqMan | 11 | 136 | 244 | 89 | 469 | 330 | 482 | 186 | 998 | 0.669 |
| Deng | 2019 | Glioma | China | Asian | HB | MassARRAY | 11 | 185 | 303 | 114 | 602 | 432 | 641 | 227 | 1300 | 0.683 |
| Gu | 2019 | Prostate | China | Asian | HB | PCR-RFLP | 12 | 383 | 473 | 159 | 1015 | 374 | 496 | 182 | 1052 | 0.426 |
| Minina | 2019 | Lung | Russia | Caucasian | PB | AS-PCR | 10 | 198 | 116 | 26 | 340 | 220 | 100 | 15 | 335 | 0.404 |
| He | 2019 | Lung | China | Asian | HB | PCR-RFLP | 7 | 88 | 94 | 28 | 210 | 61 | 142 | 80 | 283 | 0.891 |
| Ramezani | 2019 | Breast | Iran | Asian | HB | ARMS-PCR | 6 | 124 | 60 | 2 | 186 | 138 | 54 | 8 | 200 | 0.358 |
| Dantas | 2019 | Gastric | Brazil | Caucasian | HB | AS-PCR | 7 | 86 | 16 | 0 | 102 | 87 | 15 | 0 | 102 | 0.422 |
| HB, Hospital based; PB, Population based; FB, Family based; NHL, non-Hodgkin lymphoma; SCCHN, Squamous cell carcinoma of the head and neck; HNC, Head and neck cancer; OSCC, Oral squamous cell carcinoma; PCR-RFLP, Polymerase chain reaction-restriction fragment length polymorphism; APEX, Arrayed primer extension; HRM, High resolution melting; HWE, Hardy-Weinberg equilibrium. | | | | | | | | | | | | | | | | |


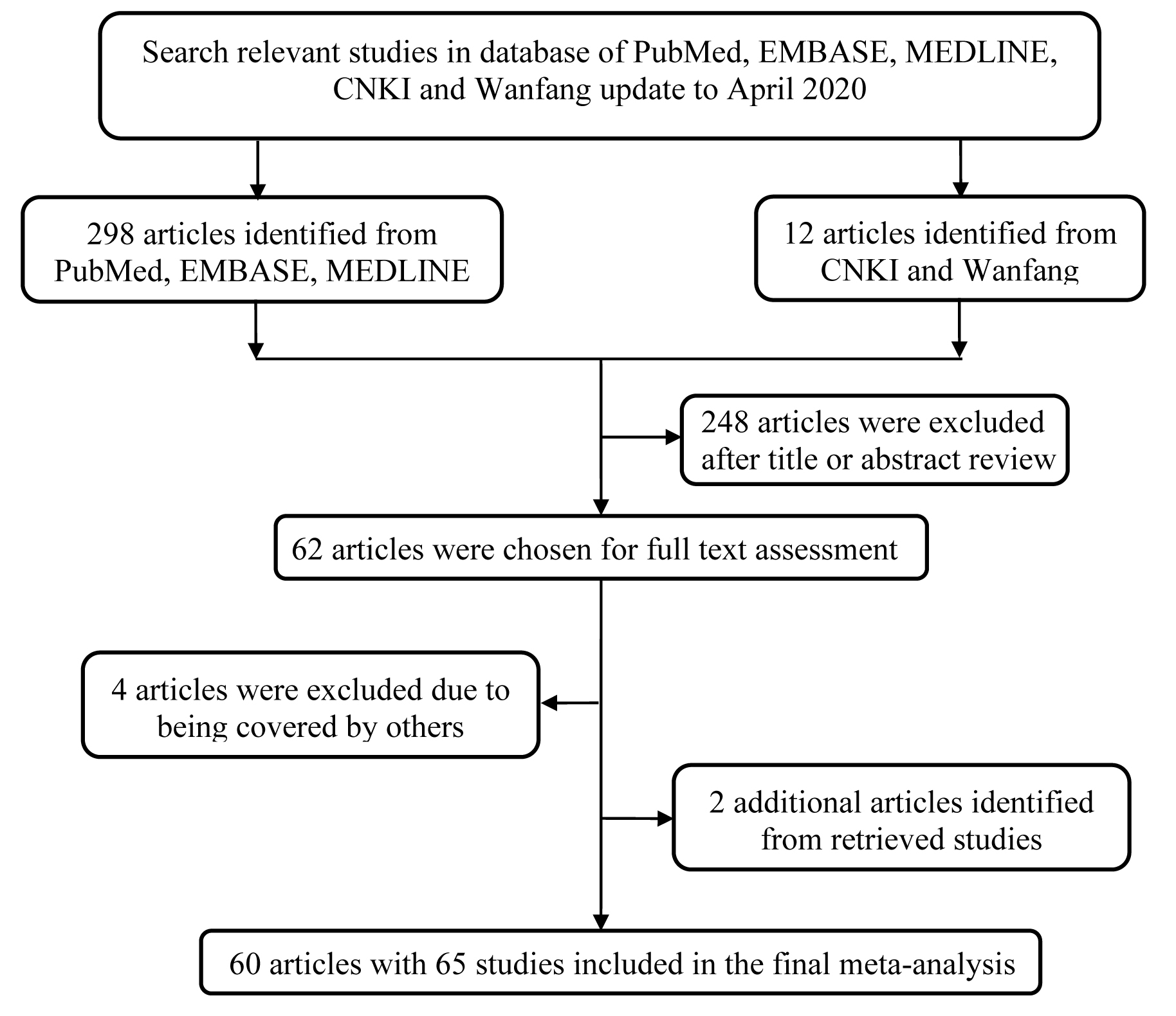


**Figure S1.** Flow diagram of the identification process of eligible studies.


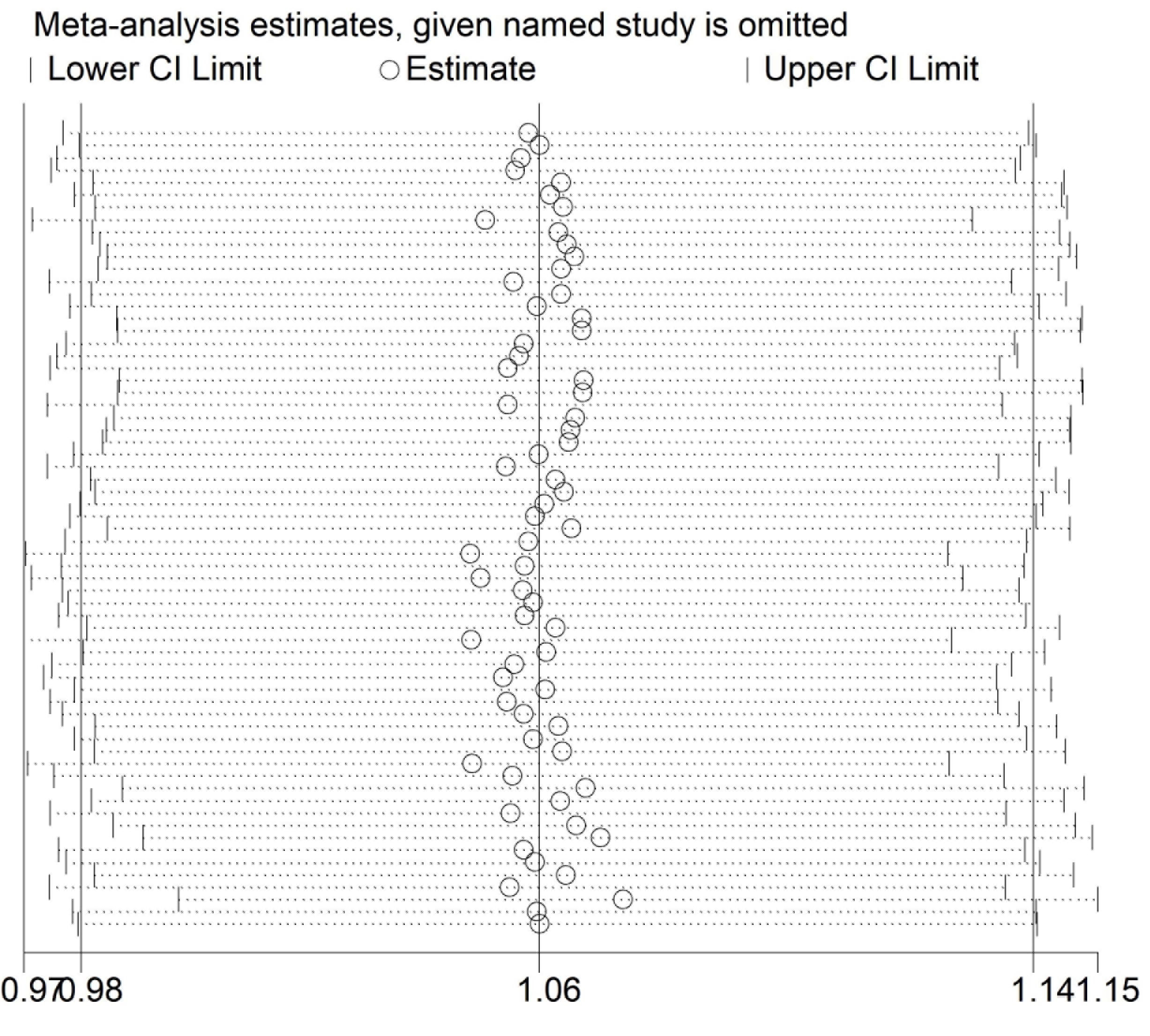


**Figure S2.** Sensitivity analysis of the association between PARP-1 rs1136410 C>T and cancer susceptibility. Each point represents the recalculated OR after deleting a separate study.


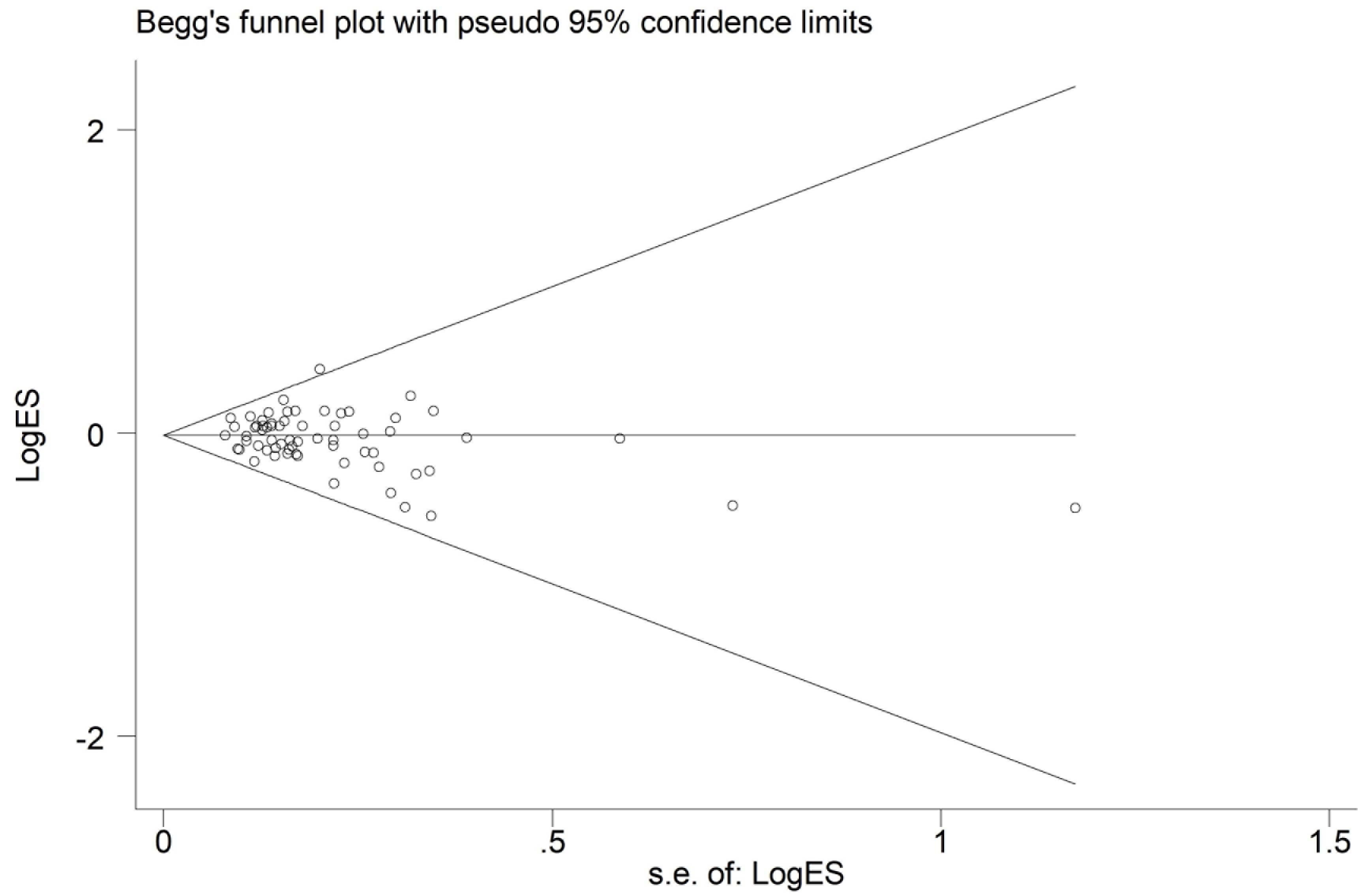


**Figure S3.** Funnel plot analysis to test publication bias for PARP-1 rs1136410 C>T polymorphism under the dominant comparison model. Each point represents a separate study.
